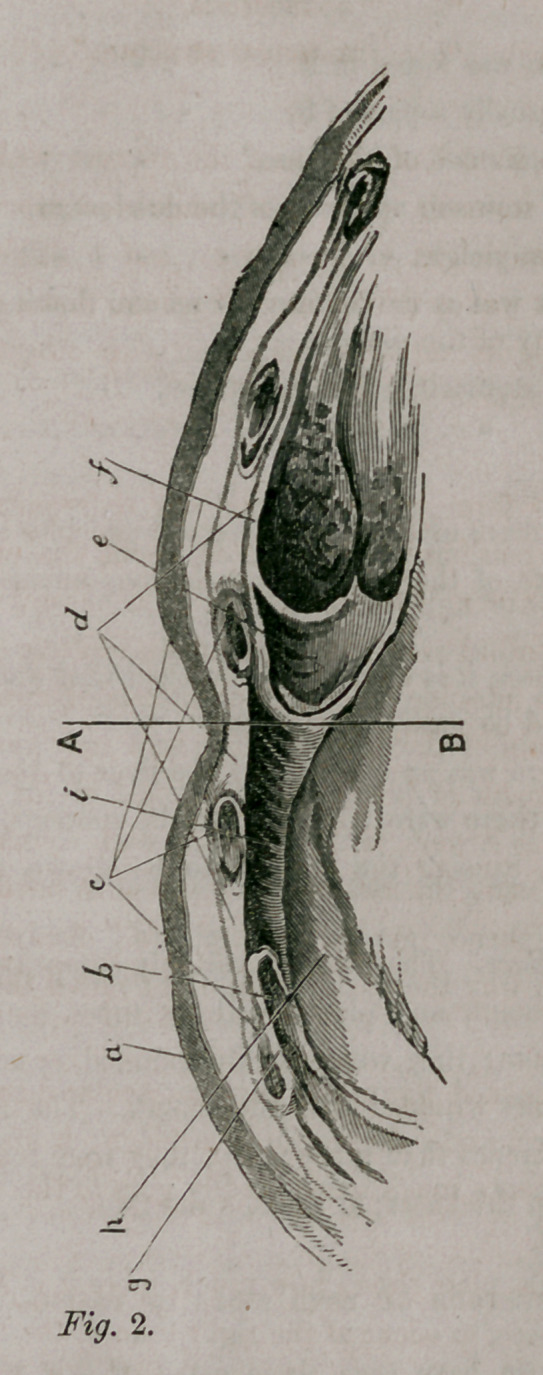# Peculiar Case of Diaphragmatic Hernia

**Published:** 1853-06

**Authors:** Henry I. Bowditch

**Affiliations:** Member of the Boston Society for Medical Observation


### BUFFALO MEDICAL JOURNAL

AND

### MONTHLY REVIEW.

VOL. 9. JUNE, 1853. NO. 1.

### ORIGINAL COMMUNICATIONS.

---

ART. I.— *Peculiar case of Diaphragmatic Hernia, in which nearly the whole of the left side of the diaphragm was wanting, so that the stomach and a great part of the intestines lay in the left pleural cavity; compressing the left lung, and forcing the heart to the right side of the sternum. This condition, evidently congenital, existed in a man who died at the Massachusetts General Hospital, with Fracture of the Spine, caused by a heavy blow upon it. To which is added an analysis of most, if not all of the cases of diaphragmatic hernia found recorded in the annals of Medical Science.* By Henry I. Bowditch, Member of the Boston Society for Medical Observation. Presented to the Society in 1847.

#### RECORD OF THE CASE.

The following, imperfect, notes of the case were obtained from the records kept by the surgeons of the hospital. I examined the youth on the day of his entrance, in order to observe the effects produced on the action of the heart by so severe an injury as fracture of the spine. I was much surprised to find the signs detailed below, and was satisfied that the intestines were in the left pleural cavity. I supposed that there had been rupture of the diaphragm at the time of the accident. On several subsequent examinations, every one, I believe, coincided with me in this diagnosis, as to the fact of the altered position of the alimentary canal. The case, I believe to be wholly *unique*, in certain particulars, viz., in the preservation of life and tolerably good health for so many years, whereas it appears from recorded cases, that death usually supervenes, either immediately after birth, or at a very early agé, in the vast majority of such cases.

*Sept.* 29, 1846. F. L., æt. 17, laborer, entered the hospital with fracture of the spine. It appeared that when a child he was surprised, on comparing his chest with those of his school-fellows, to find that his heart did not beat as theirs did, but to the right of the sternum. He had been troubled all his lifetime with palpitations of the heart, and by frequent "stitches" in the left side; and often had attacks of total unconsciousness, by which he was for some time wholly disabled.

*Sept.* 25. While resting from his work of raising a piece of timber, the derrick he had been using, broke, and fell, striking him about the middle of the back, and fracturing the spine.

The manner in which the blow was received cannot be exactly ascertained. The only facts obtained are, that he was sitting down at the time, when the derrick swayed, broke, and struck him on the back, probably not directly.

The effects of the accident were temporary loss of consciousness, paraplegia, and imperfect anæsthesia below a line, drawn around the body through the umbilicus, and severe pain in the left chest and abdomen, which diminished after two days. The accident occurred at Gloucester, where he received medical treatment. A catheter was passed twice daily, and two or three cathartics given. On the fourth day, he was brought to the hospital on a litter.

*On examination.* Intellect unaffected. Skin hot. Pulse 132. A protuberance on the back, occasioned by the spinous processes of the three lower dorsal and first lumbar vertebræ. Complete paralysis of lower extremities, with slight degree of insensibility. Fullness and dullness on percussion at hypogastrium.

Pulsation of heart natural, but entirely to the right of median line. Respiration thoracic. Right chest laboring more than left. Left chest more prominent than right, both in front and at side. On percussion, left front chest highly resonant as far as a line dropped from anterior boundary of axilla. Beyond that, dull, even on the back as far median line. Right chest natural.

*On auscultation.* No respiration over whole of left chest except from the clavicle down to the space between the second and third ribs. In its place a mixture of gurgling, whistling, and blowing sounds was heard, like those heard over the abdomen, and produced by flatus and intestinal motion. These were not generally affected by cough or inspiratory effort, though sometimes excited by either. No bronchial or amphoric sound. Metallic tinkling occasionally. Voice natural. Impulse and sounds of heart most distinct at right of sternum.

*Diagnosis.* Probably rupture of diaphragm and intestines in left chest. Catheter was passed. Elixir opii., gtt. xxx. given, and patient left for the night.

*Sept.* 30. Slept well. Had no pain. Pulse 132.

*Oct.* 1. Lies quiet; makes no complaint except of flatus. Enema; laxative diet.

*Oct.* 13. An amphoric sound, with metallic tinkling, is occasionally heard in left front chest, most intense over cartilages of fourth and fifth ribs. It seems rather stomachic than pleuritic, or pulmonic, its tone being very sharp; it is but slightly affected by the respiratory act.

*Oct.* 15. Respiration labored. Cough frequent. Throat is clogged with mucus, which he raises with great difficulty. Urinary bladder seems to contain air. It is resonant up to umbilicus, but found contracted on the catheter being used.

*Oct.* 17. Very feeble. Does not readily answer. Appetite quite good. Auscultatory sounds the same. Mouth sore.

*Oct.* 18. As yesterday. Urine passed freely by catheter. Bladder is felt in hypogastrium as a small, hard, round tumor.

The above detail of symptoms is sufficient for my purpose. The patient died Oct. 20.

The post-mortem examination was made very hurriedly, owing to circumstances beyond our control. The trunk presented no unusual appearance in front. Abdomen moderate in size, certainly not distended. On raising the sternum, the stomach, the major part of the colon, and several folds of the small intestine, with the omentum, were found in the left chest. These organs were much distended with flatus, but appeared perfectly healthy. No trace of recent lymph or injection about them on the pleura. The lung was compressed to the greatest degree and looked like a lung that had been confined by a pleuritic effusion, save that it had not the usual *sodden* aspect observed in pleurisy. The heart was pressed to the right side, but that, with the right lung, was healthy. The liver, resting upon the right side of the diaphragm, was normal. The spleen was healthy, and in its usual situation under the left ribs. The bladder was seen above the pubes, and contained about half a pint of purulent, flaky-looking, very offensive urine. A fold of small intestine was adherent to its fundus, by soft adhesions, and extended from there to the umbilicus, and was much distended with air. The coats of the bladder were dark and gangrenous. The diaphragm was perfectly healthy at the right side, but was almost wholly wanting at the left. It consisted — 1st. Of a triangular piece extending from front backward. This was 5½ inches long from sternum to spine, and only 2½ inches broad at its base, which was attached to the sternum and cartilages of ribs. Toward the spine it presented an opaque, whitish, rounded, somewhat cord-like aspect. On examination it was found composed of a muscle, and on each side was serous membrane, viz., pleura and peritoneum. Near the sternum and vertebræ, for the space of about an inch, these two membranes were united, and smoothly so, the line of demarkation in the part near the spine being invisible, while in that toward sternum they were joined by a cellular structure. The intervening space showed the muscle about ¼ inch thick, and the two membranes firmly attached to it. 2d. There was a small semilunar portion only of the diaphragm near the spleen, lying by the side and a little underneath the intestines, that had passed into the thorax. But over the whole of the breast and a good part of the side, the peritoneum and pleura seemed continuous, forming one large smooth cavity.

It was evidently a fœtal arrest of development.

### ANALYSIS OF CASES RECORDED BY AUTHORS.

The preceding case having been one of exceeding interest to me, I determined to investigate the whole subject, as I might find it displayed upon the records of medical science of the past and present (1846) times. The results of that investigation, I propose now to lay before the society.

The earliest writers of our art believed that a wound of the diaphragm must inevitably be fatal. Even the celebrated Dr. Fothergill, in a letter that he wrote to Dr. Mead about one hundred years ago, and in which he gives a very interesting account of a case of Diaphragmatic Hernia, thus exclaims: "Every one skilled in medicine, I think, will suppose, from the history, that the disease was a new one. But who would ever have conjectured that the diaphragm (septum transversum) was divided asunder, and that a large portion of the stomach and intestines had rushed through this opening into the breast?" And again, in his naive enthusiasm, he says: "Behold! a sight never, that I am aware of, seen before!"*

Unfortunately, Dr. Fothergill's learning failed him on this point, as I shall now proceed to show.

* Works of John Fothergill, &c. By John K. Lettsom. London, 1784.

A century and a half, or thereabouts, before the above letter was written, we find two cases reported in the quaint but still admirable Opera Chirurgica of the father of French Surgery, old Ambrose Parè. One of them proves the incorrectness of the above mentioned opinion of the older writers, that a wound of the diaphragm is necessarily fatal.*

Following him, I find a letter written in most barbarous Latin, by Sennertus to Fabricius Hildanus, in which a melancholy tale is told of a soldier, who, in despondency of heart, fell upon his sword. It entered about the fourth and came out at the ninth rib, wounding the diaphragm in its course; as was proved six months afterward, at which time hernia was discovered.†

Still later, comes to my notice a case of congenital disease of this kind, mentioned by Riverius. The sufferer, he tells us, was a pensive youth, 24 years of age, and one lung was almost wholly compressed in consequence of hernia of some of the organs of the abdomen.‡

Other cases appear in the Philosophical Transactions,|| Memoires de l'Academie Francaise§; but the first person who attempted a regular treatise on the subject was Kirschbaum, a little before the middle of the last century. He has collected seventeen cases from his own observation, and from the works of others. His dissertation is well worthy of the times and of the place in which it is now found; namely, in works of the "most excellent Haller." ¶

Not long after this, that extraordinary man, the pupil of Valsalva, and upon the fame of whose genius Italy might well have rested a century,—Giambattista Morgagni took, as the subject of a part of one of his letters, the question of diaphragmatic rupture. He treated it, as he always has treated his subjects of investigation, in a most accurate and manly style.**

Passing through the numerous, though briefly related and tantalizing, cases, recorded by Lieutaud,†† the interesting observations by Dr. McAulay‡‡ and others by Vicq d'Azyr|||| and Portal,§§ we come to the great surgeon

---

* Opera Chirurgica ab Ambrose Paræo. Franckfort, 1610. Ch. 30, p. 230.

† Opera Gulielmi Fabricii Hildani. Franckfort, 1646, cen. 2, obs. 33, p. 108.

‡ Lazari Riverii Opera Med. Univer. London, 1698, Obs. Cent. Quart., obs. 67.

|| Philos. Trans. Abridg. 1594 to 1702, vol. iv., 630. Sir Charles Holt.

§ Memoires de l'Academie, 1729, pages 11 and 124. Cases by Chauvet and Senac.

¶ Dissert. Chirurg.. vol. iii., p. 217. Lausanne, 1755.

** Seats and Causes of Disease, vol. iii., Letter 54.

†† Historia Anatomica Medica Auct., Joseph Lieutaud, vol. i., obs. 208, &c.

‡‡ Medical Observations and Inquiries, vol. i. London, 1771.

|||| Memoires de l'Academie Francaise, 1772. Second part, p. 81.

§§ Cour d'Anatomie Medicale, par Antoine Portal, Tom. v., p. 82. Paris, 1803.

of modern times, and his magnificent work on the general subject; I allude to Sir Astley Cooper and to his work on Hernia.* To this gentleman we owe the first systematic and thorough discussion of the various forms of this complaint, although I think that one more class may be justly added, consisting of only two recorded cases, to which I shall allude at a future time.

Since the publication of Mr. Cooper's works, many isolated cases of this complaint have been published, and may be found in the various journals of the day. Admirable treatises have been written upon it by Lawrence,† Cloquet and Berard,‡ Percy,‖ Stierling,§ Dreyfus,¶ Auzelly** and Mehliss.††

Upon 88 cases, some collected by these various authors and others found scattered through medical journals published since the commencement of the present century, I shall rest the results that I shall present. I have subjected them to a strict numerical analysis. This is the largest number ever collected for this purpose, and although some of them, especially the earlier ones, are not quite so much in detail as we could wish, I think some curious results may be obtained from the whole mass. This seems to be a small number of observations; but I know that I have carefully examined many works and journals from 1610 down to 1846, and I am convinced but few more can be found. Every one, also, who consults his own experience, must feel assured of the infrequency of this occurrence. Curling says only two cases have occurred in ten years at the London Hospital, 1000 patients being annually admitted.‡‡

---

* A Treatise on Hernia, by Astley Cooper, &c. London, 1824.

† On Ruptures. London, 5th edition. 1838.

‡ Dictionaire des Sciences Medicales, in 30 vols., 1835. Art. Diaphragm.

‖ Dictionaire des Sciences Medicales, 1818. 'Art. Diaphragm.

§ Dissertatio Inauguralis Anatomico-Chirurgica de hernia diaphragmatis cum tabulas iii. Auct. *Hub.* Griff. Stierling, Heidelberg, 1834, in 4to. See Archives Gen'les de Medecine, second series, vol. xii., p. 387.

¶ Abhandlung ueber die Brueche des Zwerchfells in beziehung auf gerichtliche arzneikunde, &c., &c. Tubingen, 1829. Journal des Progres, (vide below.)

March 15, 1847. I have not been able to procure copies of the two last mentioned, but of one of them, at least a very full account is given in the Journal des Progres des Sciences et Institutions Medicales, 1829. Paris, Tom 17, p. 125. With the results of this author I shall frequently compare my own, because his paper purports to be an analysis of facts.

** These pour le doctorat en Medecine, presentée et soutenue le 30 Août. 1842, par Aristode Raymond Auzelly.

†† Die Kraukheiten des Zwerchfells des Menschen von C. W. Mehliss, M. D. Eisleben, 1845. (B. and F. Med. Rev.)

‡‡ British and Foreign Med. Rev., May 5, 1837.

### CLASSIFICATION OF THE SUBJECT.

I propose to treat the subject under the following general heads:

1st. Anatomical Characteristics.
2d. Symptoms.
3d. Causes.
4th. Ages, sex, profession, &c., of patients.
5th. Duration of life in congenital cases and in those produced by wounds.
6th. Do. during fatal attacks.
7th. Different species of Hernia.
8th. Diagnosis.
9th. Prognosis.
10th. Treatment.

### ANATOMICAL CHARACTERISTICS.

On which side of the diaphragm was the hernial opening found?

*Table* 1.

| | | |
|---|---|---|
| It was observed in the left side of diaphragm | 41 | times. |
| " right " | 18 | " |
| " both " | 3 | " |
| Diaphragm was wanting | 1 | " |
| " and mediastinum were absent | 1 | " |
| " was pushed up into chest on one side, | 2 | " |
| Mediastinum wanting with double rupture, | 1 | " |
| Doubtful which side of diaphragm was ruptured, | 21 | " |
| | 88 | |

Why is it that rupture of the diaphragm occurs so much oftener on the left than on the right side? There are many reasons why this should happen:

1st. Among the more obvious, it may be mentioned that the great mass of the liver, partially united, as it is, to the diaphragm, becomes a kind of bulwark to defend the right side of this muscle from undue pressure.

2d. The right crus of the diaphragm is longer and stronger than that of the left side.

3d. There are two fibrous bands at the right side of the diaphragm, which do not exist at the left.

4th. We have in addition to all these means of support for the right side, the exposed condition of the left side of the diaphragm, and two minor points of interest, bearing on this subject, viz: two distinct pouches in the left side, one for the spleen, the other for the cul-de-sac of the stomach to rest in.*

These reasons appear to me more than sufficient to account for the fact that hernia of the right is less frequent than that of the left side. But great as it is, the numbers do not give so strong a view as the expression by Scheller, of Berlin, who says that "the hernia of the right side is excessively rare."†

Nevertheless, although the proportions given by my numbers may not be entirely accurate, owing to the comparatively small number of cases analyzed, they are a greater approximation to the truth than any general assertion.

EXAMINATION OF THE PECULIARITIES OF HERNIA OF THE RIGHT SIDE.

Hernia into the right side of the thorax occurred 18 times. Of these cases, 11 presented the very unusual form of hernia with a complete sac, formed by the pleura and peritoneum. So rarely has this form of the affection been observed that all writers on the subject have noticed its infrequency. Cloquet and Berard said,‡ in 1835, that in all the Annals of Medical Science, they could find but two such facts. Though they were in error, when they made the number‖ of recorded facts so small, they were right on the general proposition that sacculated diaphragmatic hernia is very seldom met with. Lawrence speaks to the same purpose.§ Sir Astley Cooper had never seen a case.¶

But a still more curious fact is this, viz: that these 11 cases of hernia of the right side compose more than five-sevenths of all the cases of sacculated hernia that can be found in the records of ancient or modern time. To make this more plain I shall give another table.

* For these various anatomical details, I am indebted to the great work by Bourgery and Jacob. Anatomie Elementaire en 20 planches. Paris, 1836.

† Archives Gen'les de Medecine, 3d Ser., vol. 18.

‡ Dict. des Sciences Medicales, 1835. Art. Diaphragm.

‖ At least five I have found in authors who wrote before 1835.

§ On Ruptures. London, 5th ed. 1838.

¶ Yet Mons. Auzelly (These pour le Doctorat en Medecine, Paris, 1842,) would limit the definition "hernie diaphragmatique" to such cases only.

*Table* 2.

| | | | | | |
|---|---|---|---|---|---|
| Hernia was found | 41 | times at the | left, and sacs existed | 3 | times. |
| " " | 18 | " | right, " | 11 | " |

The mention of an additional fact will make this tabular statement yet more prominent. 2 of the 11 cases had 2 sacs each, making 13 sacs on the right side of the diaphragm, while only 3 have existed at the left side of the median line.

The *causes* of the greater prevalence of sacs at the right side than at the left side are, I think, as follows:

1st. Just back of the ensiform cartilage, the diaphragm, where it comes in contact with the mediastinum, is thinner in its fibrous structure than elsewhere, and cellulo-vascular openings separate this fascia from the cartilages of the seventh rib.* Cloquet and Berard say: The anterior fibres, (of the diaphragm,) often leave a triangular space behind the ensiform cartilage, through which the cellular tissue of the mediastinum is continuous with that of the anterior parietes of the abdomen.† Wilson ‡ gives the following plate, (Fig. 1,) and it is illustrative of this subject. It represents the anterior part of abdominal surface of the diaphragm. 1. Section of ensiform cartilage; 2 2. Right and left portions of muscles of D.; 3. " A thin fasciculus which arises from the ensiform cartilage, leaving a small triangular space on both sides which is completed only by the serous membranes of the abdomen and chest." It is exactly at these weak points, I believe, that sacs usually commence. I have been able to make a small numerical statement in support of this idea. In 7 of the 11 cases in which sacs were found at the right side of the diaphragm, the part through which the hernia had taken place was noticed as in the following table:

*Fig.* 1.

*Table* 3.

In 2 it was "to the right of the ensiform cartilage."
2 " "just back of the ensiform cartilage."

* Bourgery and Jacobs, Elemens, &c., ut supra,

† Dictionaire des Sciences Medicales, 1835.

‡ Anatomist's Vade Mecum. London, 1840.

In 1 it was "through the anterior fibres."
1 " " "aponeurosis."
1 " " "muscular structure."

In other words, in 5-7 of the cases, the rupture took place as indicated above. I do not, however, quote these remarks as *proving* what I say, or as being of any great weight in themselves; but I believe it to be a rule of common sense, as well as of the highest reason, that a small fact sometimes becomes highly significant when conjoined with others, whereas, isolated, it is of little or no value.

2d. These sacs form gradually, and not unfrequently in the following manner: Small portions of fat, situated about the ensiform cartilage, are gradually pressed through the thin, weak spots on each side of the muscular fasciculus just described as existing back of the ensiform cartilage. Gradually, a small fold of the intestine follows after, pressing forward the fat and keeping it at the bottom of the cavity. Such sacs may vary from the size of a thimble to one capable of holding the liver, and in one case on record there were appendices, as it were, to the main sac, each containing small quantities of fat.* In some cases the sacs are represented as having passed up into the mediastinum and thence into the right pleura. And this last fact brings us to the final reason why they tend to the right rather than the left.

3d. In the annexed figure we have an ocular demonstration why, after a sac begins to form under the ensiform cartilage: it first presses into the mediastinum, and thence much more readily goes to the right than to the left side of the thorax.

A glance at this plate shows how much more it is likely for hernia, that comes on by degrees, to occur at the right than at the left. The right pleural cavity is, in fact, much nearer the median line A B, than the left one is, and 2d, the left is compressed to about one-third the size of the right.

---

* The following case by Berard, Jr., (Supplement to Scarpas,) illustrates these views: "The opening of the diaphragm was caused by the insertion of its anterior fibres into ensiform cartilage. There were two sacs, one at the right three inches long, of the size of the intestine ; other at left, size of thimble. The mouths of these were smooth and round, and a little smaller than the fundus. At the bottom of the smaller one was a small globular body of fat. The mediastinum was distended with fat, and from both sides of it were protruded several little fatty tumors like appendices epiploicæ."

*Fig.* 2.

This figure shows a horizontal section of the front of the chest on a level with the ensiform cartilage, and seen from above.

*a*, skin; *b*, muscles on the parietes of the chest; *c*, sections of cartilages, &c., of ribs; *d*, intercostal muscles; *e*, cavity of right pleura; *f*, lung collapsed; *g*, part of pericardium; *h*, left pleural cavity.* A B, median line.

*Contents of the Sac.* They were noted in six cases.

* Copied from Bourgery and Jacobs' plates. See above.

*Table 4.*

| | |
|---|---|
| The ornentum was found in it - - - - | 6 times. |
| The colon; usually a part of it, - - - - | 5 " |
| " An enormous mass of intestine," - - - | 1 " |
| Right end of stomach and part of the duodenum, - | 1 " |
| Appendices epiploicœ, - - - - - - - | 1 " |
| A mass of fat was at the bottom of one and floating in the cavity of the pleura, | 1 " |
| Part of liver, stomach, colon and spleen, - - - | 1 " |
| " " " - - - - | 1 " |

Usually one or more of these parts were found into the same sac, and in one case, were parts of the stomach, duodenum, omentum and arch of the colon.

In two of the cases, it is expressly mentioned that there was no adhesion, and the parts could be easily drawn out.

In one case, there was an adhesion of the liver to the interior of the sac; and in two more, there were adhesions to the contents, and likewise, externally, to the ribs, causing the parietes to be drawn in with each act of respiration.

*Nature of the Sacs.* They were invariably composed of the two serous membranes, peritoneum and pleura, with, at times, some thickened cellular membrane over them; they were usually rounded, or conical, smooth at the fundus, and at times wrinkled near the mouth. The mouths were usually smaller in circumference than other parts; they were rounded, like the pylorus, and variable in thickness; at times, quite thin.

### RUPTURE OF BOTH SIDES OF DIAPHRAGM.

There were, as we have seen, three cases of this kind. (Table 1.) It must be, therefore, a very rare affection, except, perhaps, in very malformed subjects.

Two of these cases were congenital, and in one the child never breathed; in the other, life was sustained ten months, but with very severe symptoms, (dyspnœa, vomiting, &c.;) and the third was that of a man who, at the storming of a certain citadel, was thrown down from a high rock and killed almost instantly.

From these facts, though small in number, we may certainly be confirmed in the idea, which would naturally arise, that with a double hernia it is impossible to have a comfortable life, and usually death supervenes immediately. At the same time, however, the second case wholly sets aside the opinion that children, born with hernia of the diaphragm, even of a most severe character, invariably die. As we proceed we shall find oth cases in confirmation of this suggestion.

### DIAPHRAGM AND MEDIASTINUM WANTING.

This is a very rare affection. Only once have I found it recorded. (Table 1.) Unfortunately this case is given, with very few details, by Lieutaud, as having been noticed previously by Diemmerbrock.* But, strange as the fact may appear, the child, thus wanting in two of the most important parts, lived to the age of seven years; always suffering, however, "from chronic asthma and frequent cough."

The mediastinum was wanting in one case, given by Sir Chas. Holt.† The child lived two months, moaning, and with constant dyspnœa. Absence of mediastinum, and hernia of the left side were observed at the autopsy.

### DIAPHRAGM PUSHED UP INTO THE CHEST.

This is likewise of very rare occurrence, only two specimens of it having been observed among the 88 cases. (Table 1.) Strictly speaking, there is no hernia in the case, the whole muscle on one side being thrust upward, and thereby compressing the corresponding lung. One was the case of a soldier to whom, after a debauch, an antimonial emetic had been given, and death was the result. At the autopsy, the diaphragm was found pressed strongly up into the chest. Of this case, however, a person may have some doubt; but I do not see that any one can doubt about that related by Senac.‡ In that, the right side of the diaphragm was greatly pushed upward, almost to the clavicle, and the right lobe of the liver was in the space. The lung, of course, must have been as much compressed, as if there had been a rupture of the diaphragm.

### ABSENCE OF THE MEDIASTINUM WITH DOUBLE HERNIA.

I have found only one case of this kind. (Table 1.) It is quoted by

---

* Historia Anatomica Medica. Paris, 1767. Vol. i., page 100, obs. 792.

† Philosoph. Trans., ut supra.

‡ Memoires de l'Academie Francaise. 1729, page 124.

Kirschbaum,* and by Lieutaud,† having been originally recorded by Becker.‡ It is one of the most extraordinary in the annals of medicine. The subject of it was a child, five years old at the time of its death, and who, from the age of two years, had had gradually augmenting dyspnœa, with forcible elevation of the chest, great liability to cough, and some dyspeptic symptoms. At death, the heart and liver lay in the right, the spleen and stomach in the left pleura. Verily it seems hardly possible for a human being to have lived, even a moment, under such circumstances, and yet, supposing that the organs were (some of them at least) thrust into their abnormal positions not immediately after birth, the symptoms distinctly showed that the major part of the disease must have been affecting the little patient for months, perhaps years, before death.

CONDITION, SITUATION, &C., OF THE OPENING IN THE DIAPHRAGM, &C. NOTICED IN SEVENTY-SIX CASES.

*Table 5.*

| | | | |
|---|---|---|---|
| It was described as | *round,* | 19 | times. |
| " | *a lunar arch,* | 4 | " |
| " | *oval,* | 1 | " |
| " | *smooth,* | 5 | " |
| " | *large,* | 19 | " |
| " | *cartilaginous,* | 2 | " |
| " | *thick, callous,* | 3 | " |
| " | *opake, yellowish, firm and even,* | 1 | " |
| " | " *muscular,* | 1 | " |
| " | *thin, soft edges,* | 1 | " |
| " | *large and recent,* | 10 | " |
| " | *edges uneven, red, fringed,* | 2 | " |
| " | " " | 1 | " |
| " | *torn from sternum and side of chest,* | 3 | " |
| " | " *attachment near œsophagus,* | 1 | " |
| " | *owing to deficiency of fibres,* | 5 | " |
| " | *through œsophageal opening,* | 3 | " |
| " | *aperture for intercostal nerves,* | 2 | " |

* Haller's Diss. Chirurg. (as above,) vol. iii., 217.

† Hist. Anatom. Med., vol. i., obs. 216.

‡ Acta Erudita Lips. A 1706, Apr., p. 17.

| | | |
|---|---|---|
| Pleura and peritoneum were united . . . . | 3 | " times. |
| Peritoneum abruptly terminated . . . . . | 1 | " |
| A valvular apparatus formed by pleura, . . . | 1 | " |
| It was in the muscular structure . . . . . . | 9 | " |
| " front of " . . . . . | 1 | " |
| " tendinous " . . . . . . | 11 | " |
| " behind sternum immediately, . . | 1 | " |
| Narrower than the sacs . . . . . . . | 1 | " |

EXAMINATION OF THE INFERENCES TO BE DRAWN FROM TABLE FIVE.

The *rounded, oval,* or *semi-circular tendency* of the openings is very manifest, (24 times.) This has been observed previously by authors, and a moment's consideration of the anatomical structure of the diaphragm will lead us to the reasons for this form. The muscular fibres run somewhat in a circular direction; hence their contraction would tend to the production of, *first,* a rounded aperture, modified, perhaps, by accidental circumstances, into the semi-circular or oval; and *second,* we learn from this structure of the diaphragm the almost total impossibility of a closure of the wound, in case of any injury of the diaphragm, causing a rupture of its coats.

The *smooth* and *polished* or *cartilaginous* and *thick* aspect, is very manifest from the table, (10 times.) In fact, I have no doubt that this number expresses too small a proportion. The long-continued, though, perhaps, gentle polishing by the vermicular movement of the alimentary canal, must inevitably tend to this result. Authors have noticed it before. It was very manifest in our case at the hospital, where there must have been less friction than in the majority of the cases. The same remarks may be made in regard to *thickness.*

In only one case of a chronic character were the edges described as *thin and soft.*

The opening is represented as large in 19 cases. Of these, 11 were the effects of recent severe injuries, such as falls, severe pressure, wounds, &c., followed either instantly, or in a few days, by death. The remainder were either congenital or of several years' duration. This would seem to indicate that even a large rupture of the diaphragm, though *eventually,* usually fatal, is not so necessarily fatal at the time of accident as one would at first sight suppose. I do not, however, lay much stress on this result, because some of the cases may not have been sufficiently explicit, in regard to the exact size, only a few of them having been measured, and the word *large* being in itself very indefinite.

It was *uneven* and *fringed* in three cases, two of which were from severe injury and from labor pains and evidently recent. The other was an old case of accident. Serious symptoms and signs of enteritis supervened, and death took place a year afterward; but there was nothing at the autopsy to explain the cause of this unevenness.

In two of the above cases, there was a bloody appearance of the edges and parts about, from the extravasation of blood.

The diaphragm had been largely torn from *the sternum and ribs* in three cases, from the parts *near œsophagus* in one. In two of these, one arising from overexertion under difficult circumstances, and the other from a fall from a cabriolet, death was instantaneous. The two others were congenital or of a year's duration, thus confirming what we have said above, that some cases, at least, may not result in instant death, even though a very extensive rupture take place.

Among the five in which there was "*a deficiency of muscular fibres*," three presented it at the posterior part, forming in one a "chasm." In the third the fibres were absent in such a way as to allow of a sac being formed. The first three were cases — 1st, of a still-born child; 2d, of one who lived three-quarters of an hour only; the last was that of an adult man.

### DILATATION OF ŒSOPHAGEAL OPENING.

The cases of *dilatation of the œsophageal opening* were very curious. They were three in number. They were all in adults; two of these were drunkards. In one, only, a small part of the ileum had entered into the chest; in two others a much larger quantity, viz., stomach, epiploon, duodenum, jejunum, and part of ileum. Unfortunately, few details are given.

### DILATATION OF AN INTERCOSTAL NERVE OPENING.

There were two cases of *dilatation of the passage made by one of the intercostal nerves.* The details are too indefinite for analysis. In both, however, the pancreas and a part of the colon were the parts that had passed into the chest, and in one the pancreatic vein was ruptured. The cause of these organs being thus forced upward is undoubtedly their position, directly underneath the hernial opening.

The statistics in regard to the *union or otherwise* of the *pleura and peritoneum* do not afford, as I think, a just idea of the usual condition of these membranes. Nor can I state any general rule in regard to it. In our hospital case, and at a hasty examination, these two membranes seemed to present a uniform smooth surface over the major part of the ribs from the sternum to the side; but on the muscular mass that extended from the sternum to spine, the two were united at both extremities for half an inch or thereabouts, and in the intervening space, they were separated by a cellular membrane. In one of the cases in the table, it was impossible to distinguish the dividing line. This was that of a child, twenty months old. On the contrary, in one of the severest and most chronic cases on record, given by Sir Astley Cooper, the peritoneum terminated abruptly at the orifice.

### ADHESIONS OF DIFFERENT ORGANS TO THE DIAPHRAGMATIC APERTURE.

The fact of adhesion is mentioned only eight times, and although this may not express the exact number, I have no doubt, from other statements, made by authors in regard to the *smoothness*, *roundness*, &c., of the aperture, that this conveys a tolerably accurate idea of what really happens. In one case, these adhesions were very strong, requiring the scalpel for their removal. In the others they seem to have been more slight.

The omentum adhered in three cases, the spleen and colon, each, once; and finally, the pleuræ were adherent around the edges of the aperture in one.

### WERE THE ORGANS STRANGULATED OR NOT AT THE OPENING?

The facts relating to this topic are twelve, of which five are described as cases having an entire freedom from strangulation, and allowing an easy return of the organs from the chest. In the other seven there was a very close grip by the aperture upon its contents; very serious effects were visible as consequences of the stricture. In all but two of them, the stomach or intestines thus caught were highly inflamed, usually dark or livid, and once gangrenous and ruptured. In two, peritonitis in various parts was observed, and a bloody fluid was found in the peritoneum.

Of the five cases in which there was no constriction, two presented nothing remarkable in the parts adjacent to the aperture, and in the remainder there was only a slight degree of inflammation, and this very limited in its extent.

But we must beware of inferring that there is no strangulation before death, when we find no great constriction after death. We all know that the abstraction of the vital force must diminish very much the constriction in cases of common internal strangulation, and as we shall be well aware, when investigating the symptoms, that almost all the patients die with symptoms of such strangulation, we may reason upon both species of hernia in the same way, and make deductions, somewhat more general, than statistical data would, at first sight, seem to warrant.

### DOES HERNIA OCCUR THE MORE FREQUENTLY THROUGH THE MUSCLES OR TENDON OF THE DIAPHRAGM?

Authors have left this question undecided, and we must do the same. For out of twenty-seven cases, in which mention is made of the part through which the opening occurred, we have as follows:

*Table* 6.

| | |
|---|---|
| Through muscular part, . . . . . | 15 times. |
| " tendonous part, . . . . . | 11 " |
| " " chiefly; a little in muscle, | 1 " |

Again,

*Table* 7.

| | ACCIDENTAL. | CONGENITAL. |
|---|---|---|
| In those where the *muscular portion* was ruptured, the hernia was . . . . . . . . | 7 times. | 2 times. |
| In those where the *tendonous structure* was ruptured, the affection was . . . . . . | 8 " | 1 time. |

From these tables I infer that the tendon and muscle are nearly equally liable to rupture from accident. The numbers, however, are too limited for very great accuracy.

WHICH OF THE ABDOMINAL ORGANS WAS MOST FREQUENTLY FOUND IN THE THORAX, AND ON WHICH SIDE DID THEY USUALLY LIE?

*Table* 8.

| | THORAX. | | |
|---|---|---|---|
| | LEFT. | RIGHT. | DOUBTFUL. |
| The stomach was found in the . . | 34 | | |
| " " . . . . | | 4 | |
| " " . . . . . | | | 6 |
| The small intestines, part of . . . | 21 | | |
| " " " . . . . . | | 4 | |
| " " " . . . . | | | 4 |
| The large intestines, . . . . . | 26 | | |
| " " . . . . . | | 5 | |
| " " . . . . . | | | 8 |
| The liver, part or whole, . . . | 8 | | |
| " " . . . . . . | | 7 | |
| The gall bladder, . . . . | | 1 | |
| The pancreas, . . . . . . | 4 | | |
| " " . . . . . | | | 2 |
| The spleen, " . . . . . . | 11 | | |
| " " . . . . . | | | 2 |
| The omentum " . . . . . . | 24 | | |
| " " . . . . . | | 3 | |
| " " . . . . . . . | | | 3 |
| The mesentery, " . . . . . | 1 | | |
| " " . . . . . . | | | 1 |
| Contents of stomach found in . . | 1 | | |
| The mass of the floating intestines, . . | 1 | | |
| " in chest, especially at R. . | 2 | 2 | |
| The kidney, . . . . . . | | 2 | |
| Blood or bloody fluid, . . . . | 7 | 1 | 1 |
| Fluid, . . . . . . . . | 1 | 2 | 1 |
| " or fetid gas, . . . . . | 1 | | |
| | 142 | 31 | 28 |

The gross results of this table amply confirm what we have previously proved, in regard to the greater frequency of rupture of the diaphragm at the left than at the left side.

But it will be useful to examine the facts less in detail. The relative frequency of hernia of the different organs is as follows, viz: Stomach, (44); large intestine, (39); omentum, (30); small intestine, (29); liver, (17); spleen, (13); pancreas, (6); mesentery, (2); kidney, (2).

This result agrees very well with that obtained by Dreyfus.* In examining 55 cases he found the stomach had penetrated into the chest 37 times; the colon, 24 times; omentum, 19 times; small intestine, 14 times; spleen, 11 times; pancreas, 8 times; duodenum, 6 times; and liver, 4.

### CONDITION OF THE VARIOUS ABDOMINAL ORGANS.

### STOMACH.

This organ was most frequently displaced, (46 times in 82 cases), but these numbers do not represent the *exact* proportions, because some of the reporters of cases have not mentioned the organ. Dreyfus makes it 37 in 55 cases.†

It was *wholly* in the chest in 32 of the cases; the greater part of it was there in 5; cul-de-sac in 4; large curvature and right end of do, and pyloric half, each once.

### CONTENTS OF THE STOMACH.

The contents were recorded eleven times. They were chiefly gaseous in seven cases, and once the stomach was said to be enormously distended by air. This flatus was mixed with some fluid in three cases, and food in one case. The organ contained a dark, fetid matter in five cases; in one of which it had acid, and in two, reddish characters. Half-digested food was found in two cases. In two, half-coagulated blood was the chief substance contained in the organ.

### SITUATION OF STOMACH.

Its *position* was, at times, much changed. In three cases it was turned up into the chest, the large curvature being bent upward toward the clavicle, and the pylorus and cardiac on a level nearly with the diaphragm. In another, the pylorus was on a level with 3d rib, the large curve toward the mediastinum; and in a fourth there was a still greater change, viz., the pylorus

* Abhandlung, &c. Journal des Progres, 1829, Tom xvii.

† Journal des Progres, &c., 1829, vol. xvii., p. 130.

was near the clavicle, while the cardiac orifice remained at the diaphragm, where in one case it was said to be constricted. In another, exactly the reverse took place, viz., the cardiac orifice was thrown up. In one case it was inverted forward, while the large curve was adherent to the left of the diaphragm. It was compressed in one case under the concave surface of the liver, and thrust to the right side in another.

Any one of these situations we can readily imagine would be liable to produce some difficulty in the digestive functions. I shall allude to this subject again when treating of the symptoms.

### INJURIES RECEIVED BY THE STOMACH.

It was wounded in two cases.

It was torn in the cul-de-sac, letting its contents into the thorax, in one. This occurred under a beating during a drunken frolic. The rupture was one and a half inches long.

It had a small semi-circular opening in one case where it was strangulated, and through this blood had oozed into the chest.

Though these numbers are few, I cannot but think that they indicate that the stomach is but rarely injured in these cases. The organ is such a conspicuous one, and has been examined so many times, that so grave a lesion as rupture could hardly have escaped notice had it existed.

### CONGESTION AND INFLAMMATION, &C.

The mucous membrane was said to be dark colored, (port wine color in one,) in five cases; and it was easily scraped off in one; sufficiently firm in another. The organ presented, on its peritoneal surface, marks of recent inflammation, where in contact with the intestines, in a case of general peritonitis. It was purplish outside, in another; and firmly adherent in a third. It seemed well, but more vessels than usual were seen under the peritoneum, in one. It was emphysemtous, toward its splenic portion, in one.

Finally, the organ was larger, paler, and thinner, in one case.

### THE ŒSOPHAGUS.

It presented a very abrupt change of its course in all the cases, (3,) in which it was noticed. In all it descended through the diaphragm as usual, but turned back toward the left to enter the abnormal aperture caused by the hernia, and to join the stomach in the chest.

It was pushed to the right side, back of the vena cava and diaphragm, in one case.

### SMALL INTESTINES.

*Contents.* They were inflated with air in six cases. The amount of air varied, but it was in great quantity so as to enormously distend the canal in three cases. Otherwise, the contents presented nothing remarkable.

*Situation.* The duodenum, in one case, was so pulled out of place, that it bent the common biliary duct and almost closed it. Of course, the changes in this respect depended entirely on the parts of the small intestine carried into the chest. The part nearest the aperture was most frequently changed in its position. Hence the duodenum was more frequently in the chest than other parts, but a great part of the convolutions were at times found there. (See table 8.)

### CONGESTION AND INFLAMMATION.

In seven cases the small intestines were represented as inflamed, &c.

In only one case, however, was there anything like general peritonitis. In all the others, there were merely lines or patches of congestion, of an acute character. In one, where a mass of colon was greatly constricted, the parts were dark, soft, and ruptured. In another there was an old partial adhesion of the intestines.

I think we may infer from these few facts, that anything like general peritonitis, to hasten the death, must be of very rare occurrence.

The colon, however, presented evidence of more serious trouble than the small intestines, a fact which coincides with our previous results. (Table 8.) We proceed now to its examination.

### COLON.

The transverse colon, or parts of it, was most commonly found in the chest.

It was said to be *distended with air*, in five cases. In one of these the walls of the intestine were thickened by the strangulation. This distention, of course, varied with the degree and point of stricture of the canal. The canal was empty and contracted in the parts below and at the stricture, and before it, dilated. In one case it was full of meconium, and in the chest. It was in a still-born child, and it was pushed between the œsophagus and aorta, carrying the mediastinum before it.

In one case, it was contracted; in another, it was twisted upon itself.

### LIVER.

This organ was noticed nineteen times.

Either one lobe, (as in three cases); or a considerable part, (as in two cases); or the whole organ, was found *in the right* cavity of the chest. Parts of the left lobe, (as in four cases); or the whole organ, (as in one case,) were found in the *left* cavity. Of these ten, six were cases of congenital hernia, and the patients were still-born, or death supervened soon after birth. In one it was the result of severe accident; the cause was doubtful in three more.

In four cases it was *much compressed* in that part that passed through the stricture; so that in two of them the parts in the thorax and abdomen seemed almost like distinct organs, united by pedicles.*

It was *strangulated, softened, and flaccid*, in three cases; its left lobe thin and flabby in one; nodulated in one; its veins and bile ducts much distended in one; thrust to the right side in another; it was pushed up into chest, without rupture of the diaphragm, in two.

All these appearances were undoubtedly owing to the rupture. It is said to have been scirrhous and gangrenous in one; nothing remarkable in others.

The effects of the changes in the form and position of the liver must at times produce very serious results; for example, such an obstruction of the gall-ducts as to produce jaundice. In the case mentioned above, the child died ten months after birth, and one of the symptoms mentioned is, " skin sometimes yellow," (see gall-bladder in this case,) and this is the sole case of the eighty-eight in which this symptom is mentioned.

### GALL-BLADDER.

This organ is mentioned in four cases. It was empty and collapsed in two, and in one of these the organ had been ruptured by a fall of thirty feet.

It was large and filled with ℥iss. thick black bile in the third case, owing to the fact that the bladder was pushed up more than usual and that the duodenum, being pulled out of its place, caused an abrupt turn and almost closure of the ductus communis choledochus. (See symptoms. Jaundice.)

In a fourth it was large, situated in thorax, thickened, and with an old cicatrix inside of it, and four calculi. The bile-ducts were thickened and lengthened.

---

* These, &c., ut supra, by Auzelly.

### THE SPLEEN.

This organ was mentioned sixteen times.

It was *torn in several directions,* and bathed in blood, in two cases; one from a severe bullet wound that penetrated the diaphragm and allowed the stomach and spleen to pass into the thorax; the other from the patient being overrun by a chaise; and in this case, the spleen and a quart of blood were found in the thorax.

It *lay lengthwise* in the lower part of the chest in one case.

It *adhered* to the diaphragmatic opening in another.

In one case it had been carried up by the stomach, and lay near the junction of the second and third ribs with the vertebral column.

With the exception of these injuries and malpositions, the organ seems to have been not abnormal. In fact, it would seem, *a priori*, as if it would be more difficult to produce any change in this organ than in the alimentary canal, and our statistics agree with our reasoning.

### THE PANCREAS.

This organ was mentioned nine times.

It lay *wholly in the chest* in two cases. One of these was after drunkenness and an emetic; the other was one of congenital opening. In the former case, the pancreatic vein had been ruptured, and the parts were bathed in blood. A *portion of the organ* was found in the thorax in three more cases, the rest of it being in the abdomen. In all the cases in which the side of the chest was mentioned, (viz., three,) it lay at the left side. These facts prove that, like the spleen, it is but rarely carried into the thorax.

It was pulled out of its usual situation in one other case, without being involved in the hernia.

It was said to be well in the two other cases.

From these facts I infer that the pancreas is rarely disturbed in any manner in this disease.

### KIDNEYS.

These organs were mentioned five times.

The right one lay partly in the chest, at the right side, in two cases. In one, it was the result of the trunk having been crushed by a heavy wagon; in the other, of congenital malformation.

In one case, it was ruptured; death having occurred from accident. In the other case, the organs were well.

Similar remarks may be made in reference to this as were made above, with regard to the pancreas and spleen. The kidneys are but rarely affected in cases of diaphragmatic hernia.

### THE OMENTUM.

This part was noticed thirty-one times; it was in the chest thirty times. (Vide table 8.)

| | |
|---|---|
| It was condensed and adherent to the stomach in . . | 2 cases. |
| " dark and adherent to the diaphragmatic opening, . . | 1 " |
| " thin and with old adhesions to do. and to sac, . . | 2 " |
| " adherent by old bands to pleura near clavicle, . . | 2 " |
| " of a vivid red color, . . . . . . . | 1 " |
| " had a purulent infiltration about it, . . . . | 1 " |
| " formed a solid cord, . . . . . . . | 1 " |
| " condensed, . . . . . . . . . . | 1 " |

Thus we see that about one-third of the cases presented some of the modifications of inflammation, evidently showing that the omentum is irritated by its frequent change of position, &c., caused by the rupture. It seems, moreover, to be a part peculiarly liable to slip into the thorax.

### THE MESOCOLON.

It was mentioned once as elongated.

I have thus examined all the organs of the abdomen that have been noticed by writers in diaphragmatic hernia. I have detailed the peculiarities, &c., of each, but there is another point of interest, viz., their combinations in the hernial sac or in the pleural cavities.

The following table gives an idea of their combinations, in the various cases analyzed, in the cavities of the pleuræ, or in the sacs, in eighty cases:

*Table* 9.

| | | |
|---|---|---|
| The stomach alone was . . . . . . | found | 10 times. |
| " and omentum . . . . | " | 8 " |
| " " and colon . . . . | " | 5 " |
| " " and small intestines . . | " | 3 " |

| | | | |
|---|---|---|---|
| The stomach, omentum, and pancreas . . . | found | 1 | time |
| " " and duodenum, spleen, liver, | " | 1 | " |
| " " small and large intestines . | " | 1 | " |
| " " duodenum and arch of colon . | " | 1 | " |
| " " colon, pancreas . . . | " | 1 | " |
| " " intestines and spleen . . | " | 1 | " |
| " " duodenum, colon, spleen, liver | " | 1 | " |
| " and duodenum and colon . . . | " | 1 | " |
| " and intestines, spleen, pancreas . . | " | 1 | " |
| " and ileum, colon, . . . . . . | " | 1 | " |
| " and small intestines and colon . . | " | 1 | " |
| " and intestines, liver . . . . . | " | 1 | " |
| " and colon . . . . . . . | " | 1 | " |
| " and intestines . . . . . . | " | 1 | " |
| " intestine and spleen . . . . . | " | 1 | " |
| " and spleen . . . . . . . | " | 1 | " |
| " " and liver . . . . . | " | 1 | " |
| " " other viscera . . . | " | 1 | " |
| The small intestines . . . . . . . | " | 4 | " |
| " " liver . . . . . . . | " | 2 | " |
| " " renal capsule . . . . . . | " | 1 | " |
| The colon . . . . . . . . . . | " | 8 | " |
| " and omentum . . . . . . | " | 3 | " |
| " and liver . . . . . . . | " | 1 | " |
| " " and pancreas . . . . . | " | 1 | " |
| "All intestines" or "floating viscera" . . . | " | 4 | " |
| Intestines with mesentery . . . . . . | " | 1 | " |
| " liver, colon, omentum . . . . | " | 1 | " |
| " " kidney . . . . . . | " | 1 | " |
| " omentum, spleen, pancreas . . . . | " | 1 | " |
| Omentum, . . . . . . . . . . | " | 2 | " |
| Spleen, . . . . . . . . . . . | " | 1 | " |
| Kidney, . . . . . . . . . . | " | 1 | " |
| Liver, . . . . . . . . . . . | " | 1 | " |
| "Viscera," . . . . . . . . . . | " | 2 | " |
| Sac containing no viscera, but fat merely, . . . | " | 1 | " |
| | | 80 | |

In general we may say, that, *of single organs* which are liable to pass into the thorax, the order is as follows: Stomach, colon, small intestines, in the proportions of 10, 7 and 1.

But the combinations are much more frequent, and the stomach in its various connections with the other viscera, stands pre-eminent.

The spleen, kidney, liver, and omentum, were each found once alone. Of these the omentum and liver were contained in sacs; and the spleen and kidney were forced there by violent accidents.

In its combinations, the stomach was conjoined with the omentum most frequently, next with the colon, and afterward with the small intestines and other organs.

The position of the stomach, colon, and small intestines, are such as explain their relative liability to hernia.

### THE ABDOMEN.

Noticed eighteen times.

This part was described as *hard and contracted* in one; *contracted* in one; and as being *flat* at the upper part of the hypogastrium, (bas ventre,) in four cases. See symptoms (abdomen.)

Some authors have noticed this subsidence of the abdomen as indicative of a loss of its usual viscera. It obviously may happen. It was not particularly manifest in our case. But it is equally evident that any unusual distention of the alimentary canal, with gas, would tend to counteract any subsidence from the hernia. The suddenness of the death, apparently, has something to do with this result, inasmuch as no time may be allowed for inflammation and consequent distention of the abdomen.

Of the six cases mentioned above, three were in accidents of a most severe character and causing instant death; a fourth was from congenital malformation of the diaphragm, and death occurred in a few hours after birth; in the fifth, a fatal result came from an attack thirty hours previous, although the hernia had been produced by a fall a year before; in the sixth alone was there any time for inflammation to commence, or probable distention to occur. In this, the man lived four days after a fall, and had had hernia, probably, for several months, owing to an injury of the same kind.

The abdomen was, on the contrary, *distended,* swollen and tight in two cases. In one of these the air was effused into the peritoneum from a hole in the colon, and the man died on the fifteenth day. Of the other, unfortunately, no symptoms are given.

It was of its usual size in one case.

The peritoneum was represented as *congested* everywhere in one case, of long standing, but which proved fatal in thirty hours after the patient had taken acidulated drink. This fact, in connection with what we have previously seen, confirms our opinion that universal peritonitis is a rare occurrence in this affection.

It was firmly united to the pleura in two cases.

*Blood was effused* in five cases. All of them were the result of falls from a height, or from the persons affected having been thrown down or run over by a carriage. In this last case it was from the vena cava being lacerated that death occurred. The quantity varied. It was slight in two; but in one, lbs. vi. were found in the hypogastrium.

### MUSCULAR SYSTEM AND UTERUS.

*Blood was effused* into the muscles in one case of a patient who had fallen.

The *uterus* presented nothing peculiar to this affection. In one case it contained full grown, entirely healthy, fœtus. The mother died from the effects of labor, which augmented all the symptoms usually attendant on diaphragmatic hernia, and to which she had been, for some time, liable.

### CONDITION OF THE ORGANS OF THE THORAX.

#### *Exterior.*

The left side of the thorax was described as *larger* than the right, in one case; in another, the thorax was more prominent generally; it was longer and narrower in a third; and compressed transversely and very prominent in front, in a fourth. No sufficient and accurate observation has been made by writers upon the subject. In the second case, there was a tearing of the diaphragm in various directions, so that the intestines were forced into both cavities. I cannot but feel that this point has been much neglected, and the probability is that we should frequently find as great a difference between the two sides of the thorax, as we find in cases of pleurisy or pneumo-thorax.

### THE LUNGS.

A glance at table 8 would satisfy any reader that the lungs must be more or less *compressed* in almost all the cases of diaphragmatic hernia. Of course, the amount of this compression will depend upon the amount of extraneous matter introduced into the chest. I have examined all the cases, and find that they may be classified as follows:

*Table* 10.

| | | |
|---|---|---|
| Very much compressed, | 8 | times. |
| Much " | 47 | " |
| Somewhat " | 24 | " |
| A little " | 5 | " |
| Not at all " | 1 | " |
| Doubtful " | 3 | " |
| | 88 | |

Whence it appears that eighty out of eighty-eight of the pulmonary organs must have been so compressed as to have been seriously interfered with in their functions. (Vide "symptoms" dyspnœa, &c.) We shall, hereafter, allude to this subject as illustrative of the causes of some of the symptoms. This compression may be said to have been almost the sole chronic difficulty which the organs had to contend with; for they are not described as, otherwise, seriously deranged. On the contrary, in some cases of extreme compression, it is stated that they could be inflated and were healthy. In only one case, was any old disease noticed. In that, the man fell gradually into phthisis, and tubercles were found in the lungs. The fact is interesting, moreover, in leading us to suspect, (we cannot be entirely sure, because all the cases are not sufficiently in detail,) that even great compression in these cases does not tend to really injure the delicate pulmonary structure. This corresponds entirely with our case, for in that the left lung seemed as healthy as if nothing had been pressing it, when in fact the stomach, colon, and small intestines had been rubbing upon it from birth.

In a few cases, (six,) the lung was described as *congested*, *œdematous*, *hepatized* or *carnified.* In one of these cases there was so much compression that the organ rested on the spine.

The lung was *adherent*, by old adhesions, in three cases. I cannot say whether this represents the ratio of all the cases. I fear it does not. I think however, we may safely infer that inflammation of, and adhesion of, the displaced abdominal organs to the lungs, &c., is not so common as one would suppose, *a priori*, they would be. And here we see the absolute necessity of having in the records of our cases, many negative statements. Had all the authors, whose cases I am analyzing, definitely stated that there was *no*

adhesion of the pleura, there would have been no doubt in the present instance.

The lungs were wounded in three cases.

Of the two lungs, the left was the most frequently and obviously compressed. At times, it was fairly laid upon the spine, but the mediastinum and heart being thrown to the opposite side, the other lung must have been but poorly able to perform its own function, much less to do a double share of duty. Hence arise the dyspnœa, &c.

Finally, in one case, the lungs were emphysematous, distended, did not collapse, and the mucous membranes were red and filled with puriform mucus. In this case the sacs were too small to produce a compression to any great amount.

### PLEURÆ.

This part was noticed twenty times.

Little is mentioned of the pleuræ; whence I think we may infer that severe general inflammation of them is rare in this complaint. They were, however, represented as more or less inflamed in five cases, in three of which some recently effused membrane was observed.

There were old adhesions in nine, but all except one was of a local character; either adhesions to an old cicatrix, or to the diaphragmatic opening, or of the omentum to the pleura, &c. In one case, however, they were so strong as to cause a drawing-in of the chest during life.

It was mentioned that there were no adhesions in six cases.

There was a *fluid* in both cavities in one; and in two more there was some in the right or the left cavity. It was bloody in one. There was a great quantity of blood effused into the pleura in four cases, three times at the right and once at the left. This was evidently caused by the severity of the accident that produced the hernia. I think that from these facts we may infer that the pleuræ were not usually much diseased, in any manner.

### THE MEDIASTINUM.

Was noticed ten times.

It was wholly absent in two cases. One was in a child who lived two months with this trouble and a hernia, which, at the time of death, consisted of all the intestines save the rectum. The patient suffered much from dyspnœa, restlessness, and finally, pined and died. The second case was that given by Lieutaud. I have already spoken of it.

It was torn, to the extent of five inches, from its attachments to the sternum in the case of the soldier, who was thrown over the ramparts during the storming of a citadel.

It was said to have been pushed aside by the hernia in four cases; but it is evident that this number does not give a correct impression if compared either with the number ten or eighty-eight, for the fact that the lungs were much compressed fifty-five times out of eighty-eight, and somewhat so twenty-four times more, proves that the mediastinum must have been pushed aside an equal number of times. In one case, it was pressed, in the form of a pouch, to the right side, between the aorta and œsophagus.

### ENSIFORM CARTILAGE.

This part was turned backward and to the right in one case, in which a sac existed at the right side of the diaphragm. It was in the person of a man æt. 60 years.

### THE THYMUS GLAND.

It was mentioned in only two cases; in one of these it was described as healthy; in the other, as having been pushed to the left side of the thorax.

### ORGANS OF CIRCULATION.

The *pericardium* contained some yellow serum in three cases. These were the only times it was noticed at all. In one case there were several ounces of fluid, but otherwise this part seemed to be but little liable to disease.

### THE HEART.

The heart was noticed thirty-one times.

Its *change of position* was the most marked, and most abnormal condition. It was thrust to the right side fourteen times; to the left five times. Position not observed in other cases. How well do these data accord with what we have observed as to the greater frequency of hernia of the left than of the right side. One class of facts supports another. But do these numbers give the relative frequency of the displacement of the heart in all the eighty-eight cases? Undoubtedly not, I think, because, according to table 10, we have seen that the lungs are very frequently and severely compressed. Now although the heart would doubtless remain in position while under the influence of some compression, it must yield under much pressure. Therefore, I think, that instead of being put out of place once in five times, it must be displaced in more than half of the cases and probably much oftener than that it will be slightly removed from its usual seat.

Its *dimensions* are represented by the terms, empty, small, contracted, in four cases; very large in two. Its right auricle was distended in one; and ventricles contained thick, black blood in the second. It is described as sufficiently large in one.

In other words, it would seem, from these statements, that any cardiac symptoms that may arise must probably come from the displacement and not from any organic change of the organ. This displacement, combined with the great compression of the lungs, would seem, *a priori*, to point to some cardiac symptoms. We shall treat of these hereafter.

### AORTA AND VEINS.

The descending *aorta* was pressed to the right side, in a case of a child in whom the intestine, full of meconium, was pushed upon the aorta and mediastinum. (See mediastinum.) This canal must, however, be very materially altered in its position, especially about its arch, by the thrusting of the heart so frequently to the right side. It seems to me that such change might be likely to produce some change in the pulse, and perhaps a difference between the two radials; but in no case was that fact mentioned.

The *vena cava* was ruptured in one case near the diaphragm, in consequence of injury from a carriage passing over the body. The *pancreatic vein* was likewise ruptured in one case, which is given by Kirschbaum, after a violent emetic had been administered to relieve drunkenness. The *umbilical vein* was much changed and very turgid in a case in which there was hernia of the right side and a considerable part of the liver had passed into the chest.

### HEAD.

This cavity has never been examined in this complaint, probably from the paucity of cerebral symptoms and the attention of physicians having never been attracted to it.

### SYMPTOMS.

In considering this subject, I shall make two divisions of the symptoms, viz:

1st. Those antecedent to the fatal attack.

2d. Those occurring during that period.

In each one of these, I shall endeavor to point out those which seem to be merely accessory and accidental, and those more important ones, which are evidently dependent upon and caused by the hernia.

### SYMPTOMS, ANTECEDENT TO THE FATAL ATTACK.

#### CEPHALIC SYMPTOMS.

*(Previous to fatal attack.)*

*A priori*, one would anticipate, perhaps, some disturbance of the cerebral functions whenever, from any sudden exertion or excitement, some interruption should take place in the even tenor of the already labored respiration. But, on consulting authors, I find less to sustain this idea than I could have anticipated. The cephalic symptoms are mentioned but twice, and these, with my own, afford no data whereby to judge the question. In our own case, the lad was liable to swoon on any violent exertion, and to be unconscious for some time. I explain it in the same way that we explain syncope in diseases of the heart.

This is the only symptom that can, by possibility, as I think, be referred to the disease. In the two other cases, there was a fetid discharge behind the ears, in one; and a depression of mind in the other described by Hildanus.*

#### PULMONARY SYMPTOMS.

*(Previous to fatal attack.)*

These symptoms were noticed in twenty-nine cases.

*Dyspnœa.* This symptom was observed in sixteen out of nineteen cases. This proves the great prevalence of this symptom, and accords very well with the fact that the lungs are so frequently compressed and the heart put out of place.

It was great in two; constant in two; the chest was forcibly elevated, with dyspnœa augmenting, till death, in one; it was worse at night, and when the clothes were wrapped closely around the trunk of the body, also when in a recumbent than when in a sitting posture, in one. This was the severest class of cases. It was milder in others, viz., like "chronic asthma," in two; constant in some degree, and very much augmented on exertion, in two; occasional, in three; sudden, evanescent, and without evident cause, in one, the patient being at other times free and easy; not great or none at all, in two.

I endeavored to classify these various degrees of dyspnœa by the lesions found after death, as it seems very natural that there would be some relation between these two ranges of facts.

---

* Op. Hildani as above.

In my first endeavor to make these comparisons, I thought I should be able to give definite results; but on more minute examination I became satisfied that only the most general and indefinate deductions could be made· Two of the cases mentioned had most serious lesions, viz., rupture of the diaphragm and absence of the mediastinum. They were in my first division, and the patients had severe dyspnœa. Generally, however, the lesions in the second class were not of so severe a character as this, and the symptoms were milder. Nevertheless, our own case is a stumbling block to nicety of diagnosis; for surely the patient could not have been very much troubled, by a complete want of the left side of the diaphragm and compression of one lung, since he was able to do a laborer's work. It is possible when our powers of diagnosis are more accurate and we recognize the disease before death, we shall be able to use this symptom as a means of nice diagnosis, more than the present record of facts allows of our doing. The actual statistics point toward what we may prove to be true some years hence. Does the numerical method allow of such prophecies? That method, as I understand it, has two objects: 1st. The rigid deduction of laws from observed facts. 2d. The suggestion of other laws which it cannot prove, but which future observation may confirm or annul. If, as numeralists, we cannot take this view, we make ourselves slaves to bare statistics, and give up our reason, thus checking that far-reaching power, which makes us men and not children.

What is the reason for the evanescent attacks of dyspnœa; and why is posture at times a relief; why is there no dyspnœa at times?

The evanescent attacks of dyspnœa, suddenly coming and at times as suddenly giving off, are to be explained, I think, by the fact that by over-exertion, injury, or certain positions, &c., a larger quantity of the intestinal canal is forced upon the lungs, or perhaps the part already in the chest becomes suddenly distended with gas. Whatever may be the circumstances, we can easily conceive that they may be very transitory in their nature.

That posture should be likely to have much influence upon such a case as our own, we can easily conceive. Gravity would, while the patient was in an erect posture, tend to relieve the symptoms by taking from the compression of the lungs, and this was the fact in the observation given by Dr. Fothergill.* It was in his case such a remarkable feature, that the little sufferer, during the ten months of its life, never could lie down after the first nap, but slept in the nurse's arms, so that if during the first sleep in a horizontal position, too great an amount of intestine fell through the large opening into

* Works, 1784. (See above.)

both pleuræ, the same might tend to fall back again while in a more erect posture. One person could not lie on his back without dyspnœa.

Finally, why is not dyspnœa constant? In the first place, it is doubtless more general than our numbers would make it. But second, there are some cases on record in which the amount of hernia is so slight as scarcely to be enough to produce pressure on the lungs; and again, in some professions, (as that of a student,) there may be such slight exertion made as that the dyspnœa will be imperceptible or of the most trivial amount. Finally, the opening may be large enough in the diaphragm to allow a free passage to the organs that are in the hernia. Hence, sometimes, there would be compression; and at others, entire freedom of the thoracic organs.

### COUGH.

*(Previous to fatal attack.)*

This symptom is mentioned six times.

It was represented as either frequent, or as nearly constant from the time of the accident or from birth, in congenital cases. It was spoken of as dry in one case. I cannot believe that this fairly represents the relative prevalence of this symptom. However, from our facts, we cannot deduce more.

A slight expectoration is mentioned once.

### PAINS IN THE CHEST.

*(Previous to fatal attack.)*

These were noticed in five cases.

They were always in these cases in the left side, and in one of them they extended to the shoulder. In this last, a full meal aggravated it, and in one of the others it came on and disappeared so suddenly that it was supposed to be spasmodic. In all these cases, the opening in the diaphragm was at the left side. The symptom did not seem connected with any apertures in the diaphragm of peculiar size or shape, nor with the amount of abdominal viscera in the chest. The colon was displaced in all the cases, alone and only to the size of the fist, in one; it was connected with omentum in another, with the stomach in the third, and with the stomach and omentum in two more.

### PECULIAR SYMPTOM.

*(Previous to fatal attack.)*

In the case mentioned by Sir Charles Holt,* there was an appearance of a very peculiar character, viz: "an odd sort of working of the breast, a crawling around the ribs of both sides as if a knot of worms were there." In this case, there was a congenital, hernial opening on the left side, and the mediastinum was wanting.

I presume this motion was caused by the vermicular movements of the alimentary canal within the chest. It has some analogy to what our patient observed, who felt air pass, at times, from a spot high up on the left breast down to the pubes. This symptom is well worth attention as a means of diagnosis, and it becomes of more importance, as we may sometimes excite it, by allowing the patient to swallow while we are auscultating.

### FITS OF SUFFOCATION.

*(Previous to fatal attack.)*

These occurred, in one case, viz., that of a child who lived ten months. It was, in fact, a kind of access of dyspnœa. While nursing, the little patient would fall into a violent fit of passion. The crying and extra-exertion would produce an access of suffocation which, from its severe influence upon the whole system, instantly subdued the temper of the child, by causing physical prostration.

### CARDIAC SYMPTOMS.

*(Previous to fatal attack.)*

The pulse was always "disturbed, small and tremulous," and very rapid, in the only case in which it was mentioned.

The heart beat to the right of the sternum in one case. These data evidently give no accurate results. For a discussion of this point, see article "Pulse during attack."

### ABDOMINAL SYMPTOMS.

*(Previous to fatal attack.)*

Noticed thirteen times.

---

* Philosoph. Transactions, vol. from 1694 to 1702.

*Vomiting.* This was observed seven times; the tendencies usually commenced after the injury or soon after birth, and continued, with more or less liability on the part of the patient, until the fatal attack. It was augmented in three cases, by over-eating; and in one case, even the smell of food, or acescent food, tended to produce it.

The matters vomited are not mentioned, save in one case. In that, (a nursing child was the patient,) it was a kind of fetid, purulent pap.

In all these cases the stomach was found either wholly, or in part, in the chest. (See symptoms during attack, article vomiting.)

The other *stomachic* symptoms were as follows: weakness of it in one; liability to oppression in two; troubled by acescent food in one; dyspepsia in one; nausea and desire to vomit in one; fullness after eating, one.

These combined with the vomiting, make ten cases, out of the thirteen in which the abdominal symptoms were observed, and they seem to me to prove that the stomach is very frequently the sufferer in diaphragmatic hernia. This will be still more evident when we examine the same subject, as it relates to the symptoms during the fatal attack.

*Abdominal pains* or *colics* were noted in nine cases, or in three-fourths of the cases in which any abdominal symptoms were observed. They were usually of a violent character, and lasting from birth or the time of injury; occurring at irregular intervals, and were frequently brought on by excess in eating. They were so particularly liable to affect a worthy soldier, described by Ambrose Parè, that he was obliged wholly to forego his wonted 9 o'clock supper, after his apparent recovery from a bullet wound in the thorax. They seem generally to have been felt at the upper part of the abdomen near the diaphragm, or they were referred to the stomach. In only one case was it in the left hypochondrium. I think we may regard this sign as an important one for the future diagnosis of any case.

I endeavored to learn whether any particular state of the diaphragmatic affection, or of the parts in the hernia, would account for these pains. I found that in four of the cases, in all of which the colics were severe, the apertures in the diaphragm were small. In two more, the opening was possibly a little larger, one being two and a half inches in diameter; the other, having the œsophageal aperture opened. In one case the liver and stomach &c., were in both sides of the chest; in a second, the stomach and omentum were condensed into a ball inside of the thorax; and in the third, the diaphragm had been torn from the spine near one of its crusa.

Perhaps at a later period of the paper, in the article "Pains, &c., during fatal attack," we may be better able to decide this question.

The other abdominal symptoms were as follows: a *dragging sensation,* as of something attached to the right side and referred to the region of the stomach. This was noticed in one case, in which the stomach was thrust to the right side of the abdomen. "*Enteritis*" occurred in one case, in which a man fell from a great height, and recovered after suffering some time from this disease. Another person had an "abdominal difficulty;" probably, tuberculosis. One was described as having attacks of strangulated intestine; and of two remaining, one had *hiarrhœa;* the other, *costiveness.* It is plain that we can deduce only the most general conclusions from these isolated facts.

### GENERAL STATE OF BODY, SKIN, &C.

*(Previous to fatal attack.)*

The skin was wrinkled, and sometimes yellow, in one case of a child aged ten months, affected with congenital hernia, and in whom the common bile duct was almost closed by a change of position of the duodenum. (See gall-bladder, page —.) There was a vesicular eruption, (accidental, I presume,) about the mouth, in another.

The lips were of a violet hue, and cheeks of a deep red, in two congenital cases, in which death occurred after two and ten months; the patients were always restless and uneasy, and, in one, accompanied with much pining until death. One was "weakly and little."

In one case, a man, ten years after an accident, fell into phthisis and hectic symptoms. In the case of one man, it was said that though unhealthy he was able to work at his trade of mason. One, only, was said to be *well.*

The details of this case, however, as given by Morgangi, are scarcely sufficient to allow us to lay much stress on it. But our own case is a proof that the greatest degree of hernia may exist on one side and the patient may be *muscular* and able to do the hardest work, though liable to access of dyspnœa, &c. In two of the cases recorded, the patients were said to be very muscular. This fact is quite in accordance with what is seen in other conditions, where the disease consists of a merely local disturbance without the constitution being necessarily affected.

Table 14 supports this idea; for out of twenty-five cases in which the occupations of the patients are given, twenty-four were engaged in business, requiring activity and exertion of strength.

I find, moreover, that of fifteen individuals of whom any mention is made of the amount of development of the muscular and adipose texture, there were of women, five either fat or inclined to be so, only two thin; of men five were robust and muscular, and only one thin. (It may be remarked that this last was the only case in which phthisis was discovered); of children, three, and all of them were thin. It seems to me that from these facts we may infer that, although the patients suffering from diaphragmatic hernia may have troublesome, and at times, dangerous symptoms, nevertheless, when they arrive at adult age, there is nothing to prevent a full development of the muscular and adipose tissues.

(Concluded next month.)

## Figures and Tables

**Fig. 1. f1:**
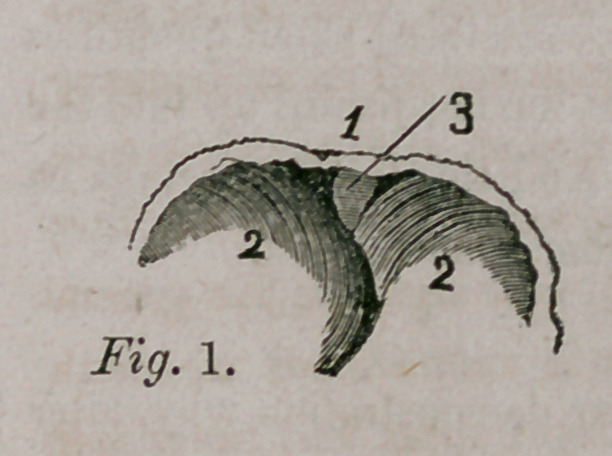


**Fig. 2. f2:**